# The effect of body mass index and preoperative weight loss in people with obesity on postoperative outcomes to 6 months following total hip or knee arthroplasty: a retrospective study

**DOI:** 10.1186/s42836-023-00203-5

**Published:** 2023-10-01

**Authors:** Natalie Pavlovic, Ian A. Harris, Robert Boland, Bernadette Brady, Furkan Genel, Justine Naylor

**Affiliations:** 1 South Western Sydney Clinical School, Faculty of Medicine and Health, University of New South Wales, Sydney, NSW 2170 Australia; 2 Fairfield Hospital, South Western Sydney Local Health District, Sydney, NSW 2176 Australia; 3 Whitlam Orthopaedic Research Centre, Ingham Institute for Applied Medical Research, Sydney, NSW 2170 Australia; 4 School of Clinical Medicine, UNSW Medicine and Health, University of New South Wales, Sydney, NSW 2052 Australia; 5 Faculty of Medicine and Health, The University of Sydney, Sydney, NSW 2006 Australia; 6 Liverpool Hospital, South Western Sydney Local Health District, Sydney, NSW 2170 Australia; 7 School of Health Sciences, Western Sydney University, Sydney, NSW 2560 Australia; 8 Faculty of Medicine and Health, St George and Sutherland Clinical School, University of New South Wales, Sydney, NSW 2217 Australia

**Keywords:** Osteoarthritis, Obesity, Body mass index, Arthroplasty, Replacement, Knee, Arthroplasty, Replacement, Hip, Postoperative complications, Patient reported outcome measures, Retrospective studies

## Abstract

**Background:**

Few studies have investigated the association between obesity, preoperative weight loss and postoperative outcomes beyond 30- and 90-days post-arthroplasty. This study investigated whether body mass index (BMI) and preoperative weight loss in people with obesity predict postoperative complications and patient-reported outcomes 6 months following total knee or hip arthroplasty.

**Methods:**

Two independent, prospectively collected datasets of people undergoing primary total knee or hip arthroplasty for osteoarthritis between January 2013 and June 2018 at two public hospitals were merged. First, the sample was grouped into BMI categories, < 35 kg/m^2^ and ≥ 35 kg/m^2^. Subgroup analysis was completed separately for hips and knees. Second, a sample of people with BMI ≥ 30 kg/m^2^ was stratified into participants who did or did not lose ≥ 5% of their baseline weight preoperatively. The presence of postoperative complications, Oxford Hip Score, Oxford Knee Score, EuroQol Visual Analogue Scale and patient-rated improvement 6 months post-surgery were compared using unadjusted and adjusted techniques.

**Results:**

From 3,552 and 9,562 patients identified from the datasets, 1,337 were included in the analysis after merging. After adjustment for covariates, there was no difference in postoperative complication rate to 6 months post-surgery according to BMI category (OR 1.0, 95%CI 0.8–1.4, *P* = 0.8) or preoperative weight loss (OR 1.1, 95%CI 0.7–1.8, *P* = 0.7). There was no between-group difference according to BMI or preoperative weight change for any patient-reported outcomes 6 months post-surgery.

**Conclusion:**

Preoperative BMI or a 5% reduction in preoperative BMI in people with obesity was not associated with postoperative outcomes to 6 months following total knee or hip arthroplasty.

**Supplementary Information:**

The online version contains supplementary material available at 10.1186/s42836-023-00203-5.

## Background

Arthroplasty is often recommended for end-stage osteoarthritis that is unresponsive to pain medication, physiotherapy and lifestyle modification. According to the Australian Orthopaedic Association National Joint Replacement Registry (AOANJRR), 97.7% and 88.3% of the primary total knee (TKA) and hip arthroplasty (THA) procedures respectively, are performed for osteoarthritis [1]. Of those, 57.5% of TKA and 39.5% of THA recipients are classified with obesity at the time of surgery [1]. Concerningly, obesity has been identified as a potential contributing factor to a greater rate of post-arthroplasty complications, including infection [2, 3], poor wound outcomes [4] and pulmonary emboli [5]. Furthermore, compared to people with a normal body mass index (BMI), obesity is associated with worse long-term patient-reported functional outcomes [2, 6], reduced mobility [6] and inadequate physical activity levels following THA or TKA [7].

Many studies have investigated the association between obesity, defined by BMI, and postoperative complications. Overall, the literature is conflicting. Some studies demonstrated no association between obesity and postoperative complications [8–10] while other studies showed that complication rates rose with increasing BMI [4, 11–16]. In particular, people with morbid obesity experience significantly greater rates of superficial and deep infection, sepsis, reoperation and readmission than those classified as overweight or having obesity [4, 13]. Regarding the evidence linking obesity with greater postoperative complications in the short-term, most studies have been retrospective in design [4, 5, 16–19] and few have investigated this association beyond 30- or 90-days post-surgery [18, 19]. Further, little is known about the effect of preoperative weight loss in people with obesity undergoing TKA or THA on postoperative outcomes. While retrospective, this study of prospectively collected data will provide a more complete perspective regarding the incidence of postoperative complications among people with obesity undergoing primary TKA or THA, their association with patient-reported outcomes to 6-months post-surgery, and whether weight loss preoperatively among those with obesity predicts patient outcomes. Thus, results from this study can be used to inform further research regarding the potential benefits of preoperative weight-loss interventions and postoperative outcomes.

## Methods

### Study design

A retrospective study of prospectively collected data from two Australian public hospital cohorts undergoing primary TKA or THA was conducted. Data were obtained from two independent clinical databases, the Arthroplasty Clinical Outcomes Registry National (ACORN) and Osteoarthritis Chronic Care Program (OACCP) [20, 21]. Ethics approval was obtained from the South Western Sydney Local Health District (SWSLHD) Human Research Ethics Committee (2020/ETH01867).

### Recruitment and screening

Eligible participants were identified from data collected between January 2013 and June 2018 inclusive. The cohort included adults with a primary diagnosis of osteoarthritis who underwent primary elective THA or TKA (unilateral or bilateral) and could be matched between the two datasets during this period. The second joint for an individual who appeared more than once was excluded so that all admissions were for one joint only. Individuals who elected to opt out were not included in the dataset.

### Data source and extraction

#### ACORN data collection

The ACORN database collected data from patients undergoing TKA or THA at multiple Australian public and private hospitals. Data were captured at three-time points only: prior to surgery, on discharge from the hospital, and 6 months post-surgery. Preoperative demographic, anthropometric, and comorbidity data, along with patient-reported outcome measures (Oxford Knee or Hip Score and the EuroQol Visual Analogue Scale [EQ-VAS]), were collected directly from patients within 2–6 weeks before surgery and their medical records by site coordinators. Acute care data, including the number and type of postoperative complications, were extracted from the medical record. Outcomes post-discharge to 6 months following surgery were collected from the patient by telephone by ACORN research officers. Outcomes included complications, the number and reasons for readmission to 6 months post-surgery, the Oxford Knee or Hip Score, EQ-VAS and the patient-rated improvement from surgery. Visits to the emergency department were collected to capture complication rates and were not categorized as readmission. Thus, visits to the emergency department were recorded as complications. The ACORN database uses an opt-out consent process. Data were collected from consecutive patients who underwent surgery. Those who elected to opt-out or who did not have surgery were not included in the dataset. Non-English-speaking patients were included in the dataset and completed translated outcome measures, where available, or were assisted by a nominated carer. This approach was shown to be reliable when compared to using healthcare interpreters for the administration of patient surveys following arthroplasty in patients with limited English proficiency [22].

#### OACCP data collection

The OACCP is a program across NSW public hospitals designed to improve the coordination of care and conservative management of individuals with osteoarthritis wait-listed for surgery [20]. OACCP data for this study were obtained from assessments conducted by clinical staff at two public hospitals within SWSLHD. Assessments were conducted on admission to the waitlist (baseline) and three months after the initial assessment. Some participants were reviewed 6 and 12 months after their initial assessment if indicated according to their needs or risk category. The following data were collected: anthropometric measures (age, weight, height, BMI, and waist and hip circumferences), comorbidities, highest level of education, language spoken at home and patient-reported outcome measures (the Knee Injury and Osteoarthritis Outcome Score (KOOS) and the Hip Injury and Osteoarthritis Outcome Score (HOOS)). Data were collected from consecutive patients. The KOOS and HOOS data were used to describe the sample at baseline but were not used in data analysis. There were no exclusion criteria for the OACCP database.

### Outcomes of interest

The primary outcome of interest was the presence or absence of postoperative complications to 6 months post-surgery. The types of complications included in the analysis are described in Table 1. The secondary outcomes of interest were patient-reported outcome measures at 6 months post-surgery: Oxford Knee Score or Oxford Hip Score, the EQ-VAS, and patient-rated improvement from surgery. The Oxford Knee Score and Oxford Hip Score are questionnaires that evaluate patient perceptions of joint pain and function experienced during the previous four weeks [23]. Patient perception regarding their general health status was assessed with the EQ-VAS. Participants rated their overall health on a 0 to 100 mm visual analog scale ranging from “worst possible” (0) to “best possible” health (100). Patient-rated improvement from surgery was assessed by the question “Overall, how are the problems now with your hip/knee compared to before your operation” and responses were measured on a Likert scale (“much worse”, “a little worse”, “about the same”, “a little better”, and “much better”). This question was based on questions measuring the patient’s perceived satisfaction and success of surgery used by the National Joint Registry in England and Wales [21].

**Table 1:** Types of postoperative complications collected for analysis

**Acute postoperative complications** (From the point of surgery until discharge from the acute hospital)	**Postoperative complications to 6 months** (From discharge from the acute hospital admission until 6 months post-surgery)
Drug reaction Delirium SSI requiring oral antibiotics SSI requiring IV antibiotics SSI requiring surgery with no prosthesis removal SSI requiring surgery with prosthesis removal DVT PE Fat emboli Respiratory infection CVS Dislocation Fracture Nerve injury Bladder infection Bladder retention Wound dehiscence Reoperation during index admission Pressure area Fall Hypotension Cellulitis Superficial SSI, earlier forms Deep SSI, earlier forms Bladder infection/retention, earlier forms Death Other	Readmission due to operated joint DVT PE MUA Dislocation SSI Other Readmission due to non-joint reasons Cardiac Kidney Cancer Other Revision surgery SSI requiring surgery with no prosthesis removal Dislocation Joint stiffness Other Postoperative complications not requiring readmission SSI requiring oral antibiotics SSI requiring IV antibiotics DVT index leg DVT other leg DVT both legs PE Dislocation Joint stiffness Bladder infection/retention Fracture Unexpected pain Cardiac Stroke Leg length discrepancy Joint or lower limb swelling Parasthesia/numbness Cellulitis Neuropathy Muscle weakness Respiratory infection Other

*SSI* surgical site infection, *DVT* deep vein thrombosis, *PE* pulmonary embolus, *MUA* manipulation under anaesthetic, *CVS* cardiovascular

### Exposures of interest

The main exposure of interest in this study was BMI stratified according to two categories: BMI < 35 kg/m^2^ and BMI ≥ 35 kg/m^2^. A BMI ≥ 35 kg/m^2^, was chosen as the cut-off based on evidence demonstrating a greater incidence of postoperative complications in people undergoing TKA or THA with BMIs greater than 35 kg/m^2^ compared to those with BMIs ranging from 30–34.99 kg/m^2^ [14, 15].

The secondary exposure of interest was the amount of preoperative weight loss in a sub-group of the sample consisting of participants with obesity (BMI ≥ 30 kg/m^2^) stratified into two categories: participants who lost 5% or more of their baseline weight preoperatively and those who did not. Preoperative weight change was calculated by subtracting the earliest recorded BMI in the OACCP dataset from the pre-surgical BMI recorded in the ACORN dataset. A minimum of 5% weight loss was chosen based on current literature which demonstrated clinically meaningful benefits for pain reduction [24, 25] and improvement of obesity-related comorbidities such as glycemia [26]. Furthermore, existing data from the OACCP at Fairfield Hospital indicated that approximately 10–15% of patients lost 5% of their baseline weight before surgery. Data regarding how and why patients lost weight before surgery was not collected. As part of the OACCP, patients received general weight management advice from a nurse and physiotherapist. There was no dietitian input at the two hospitals from which the data is obtained. Weight loss was not a requirement for surgery. Due to the small number of people with BMI ≥ 35 kg/m^2^ losing weight before surgery and the subsequent impact on power to detect a significant difference between groups, a threshold of BMI ≥ 30 kg/m^2^ was used in this analysis.

### Covariates

The following variables were identified a-priori as potential confounders to the association between BMI or weight loss (BMI reduction) and outcomes: age, sex, surgery type (TKA or THA), unilateral or bilateral procedure, American Society of Anaesthesiology (ASA) score, comorbidities (hypertension, diabetes, heart disease, lung disease, stomach or gastrointestinal conditions, renal failure, liver disease, neurological condition and depression or anxiety) and musculoskeletal conditions including low back pain and other lower limb joint problems that interfere with mobility. The inclusion of these covariates was based on established research showing that these variables may be associated with the outcomes of interest [4, 5, 11–13, 16]. Pre-surgical Oxford scores, education level and language spoken at home were also used as covariates in adjusted analyses where patient-reported outcome measures were the outcome of interest. The decision to include these covariates was based on current literature demonstrating worse patient-reported outcomes in populations with lower pre-surgical scores [27, 28] and education level [29]. Language spoken at home was used as a surrogate for cultural background based on research revealing an association between non-Caucasian populations and worse patient-reported outcomes [30]. Age and Oxford scores were measured as continuous variables while sex, surgery type, ASA score, comorbidities, education level and language spoken at home (stratified into either “English” or “other”) were treated as categorical variables.

### Sample size

The planned analysis was based on an estimated sample size of approximately 3,000 cases from the merged ACORN and OACCP datasets. For the primary analysis regarding the association between BMI and postoperative complications, it was anticipated that approximately 30% of cases would have a complication based on local data [31]. A sample size of 353 cases per group would have 80% statistical power to detect a statistically significant (alpha = 0.05, 2-tailed) difference between groups of 10% (30% in the BMI < 35 kg/m^2^ group vs. 40% in the BMI ≥ 35 kg/m^2^ group), excluding confounding variables.

### Statistical analysis

Descriptive statistics (mean, standard deviation, percentages) were used to report the characteristics of the cohort and primary and secondary outcomes. Records with missing data and invalid scores (e.g., Oxford score > 48) were removed.

Binary logistic regression was used to determine whether a BMI ≥ 35 kg/m^2^ was associated with postoperative complications in the acute postoperative period (from surgery to discharge from hospital) and to 6 months post-surgery (separate analyses). The analyses included all complications listed in Table 1 and results were adjusted for the covariates. Sensitivity analysis was completed using only major complications defined as: deep surgical site infection, deep vein thrombosis, pulmonary embolism, respiratory infection, fracture, dislocation, stroke, cardiac complications, revision surgery, readmission, and death. Furthermore, because the effect of obesity depends on the operated joint, a subgroup analysis was completed to investigate the association between BMI and postoperative complications separately for hips and knees.

Binary logistic regression was also used to determine the association between preoperative weight loss and postoperative complications to 6 months in people with obesity. Sensitivity analyses were completed for major complications in the acute period and to 6 months post-surgery separately. Covariates were included to adjust for possible confounding factors. Separate analysis according to type of arthroplasty was completed for TKA, but not THA due to the small sample size in the sub-sample.

Linear regression was used to determine the association between Oxford and EQ-VAS scores for both exposures of interest. Ordinal logistic regression was planned for analysis of the patient-rated improvement from surgery. However, because the sample was not adequately powered, binary logistic regression was used with the category denoted ‘much better’ on the 5-point Likert scale compared to all other responses.

The final regression models were achieved by including the following covariates: age, sex, surgery type, ASA and all comorbidity covariates. For patient-reported outcomes, education level, whether participants spoke English and pre-surgical Oxford score were included in the models in addition to the aforementioned covariates.

The logistic models were assessed using the Hosmer and Lemeshow test for goodness of fit and the area under the receiver operating characteristic (ROC) curve (Additional file 1). The following classification was used for the area under the curve for the ROC: no discrimination (< 0.5), poor discrimination (0.5–0.69), acceptable discrimination (0.7–0.79), excellent discrimination (0.8–0.89) and outstanding discrimination (≥ 0.9) [32]. The linear regression models were assessed using *R*^2^, the residuals vs. fitted values plot, the quantile–quantile (Q-Q) plot, the scale-location plot, and Cook’s distance plot. All statistical analyses were performed with R Environment for Statistical Computing (version 4.2.0) [33].

## Results

### Screening and Recruitment

**Fig. 1:**
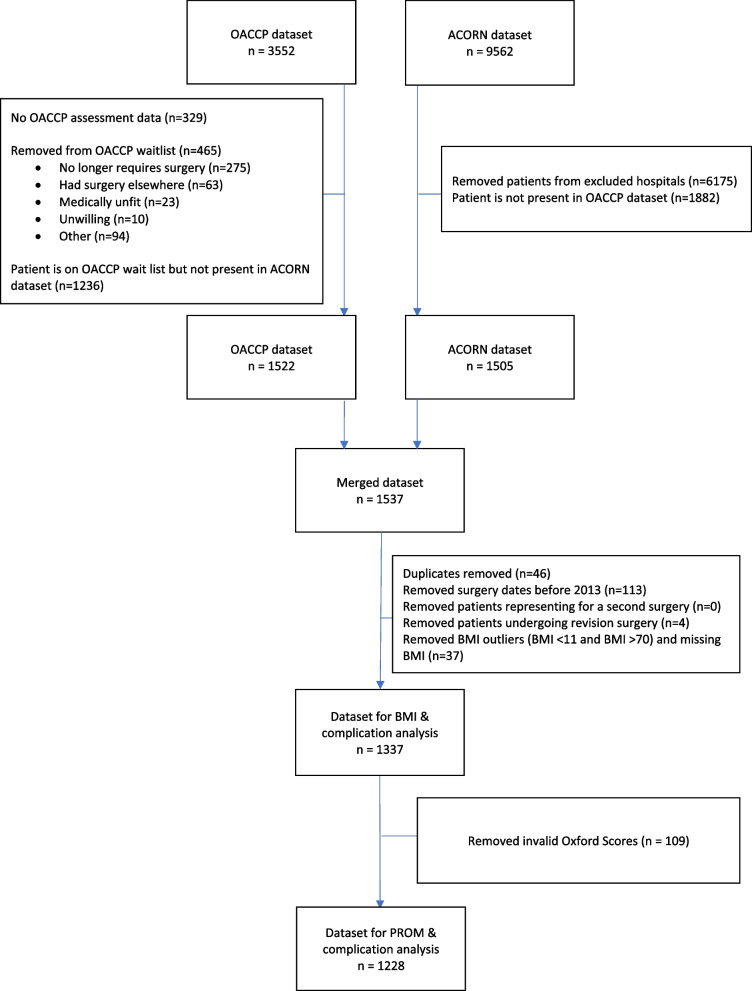
Sample derivation flow chart

Sample derivation is shown in Fig. 1. A total of 3,552 and 9,562 patients were identified in the OACCP and ACORN databases respectively. After merging the datasets and applying the inclusion and exclusion criteria, 1,337 participants with complete data remained for the primary analysis of the association between BMI and postoperative complications. Following the removal of invalid Oxford scores, 1,228 participants were retained for the secondary analysis of the association between BMI and patient-reported outcome measures. Participants with BMI under 30 kg/m^2^ were then removed to create the sub-sample of participants with obesity for analysis of preoperative weight change with respect to postoperative complications (*n* = 809) and patient-reported outcome measures (*n* = 747).

### Participant characteristics

Baseline characteristics for the overall sample (*n* = 1337), including the division between BMI category and type of arthroplasty, and the sub-sample of participants with obesity (*n* = 809) are listed in Tables 2 and 3, respectively.

**Table 2:** Baseline characteristics for the overall sample

	**Overall** **(*n* = 1,337)**	**THA** **(*n* = 399)**	**TKA** **(*n* = 938)**	**BMI < 35** **(*n* = 882)**	**BMI ≥ 35** **(*n* = 455)**
Gender, *n* (%)					
Female	849 (63.5)	229 (57.4)	620 (66.1)	518 (58.7)	331 (72.7)
Male	488 (36.5)	170 (42.6)	318 (33.9)	364 (41.3)	124 (27.3)
Age, years, mean (SD)	66.8 (9.89)	65.3 (11.8)	67.5 (8.89)	68.1 (10.1)	64.3 (9.05)
Baseline BMI, (kg/m^2^), mean (SD)	32.8 (7.12)	30.5 (6.24)	33.8 (7.25)	28.8 (3.98)	40.5 (5.31)
Surgery type, *n* (%)					
THA	399 (29.8)	399 (100)	0 (0)	309 (35)	90 (19.8)
TKA	938 (70.2)	0 (0)	938 (100)	573 (65)	365 (80.2)
Unilateral	1,316 (98.4)	396 (99.2)	920 (98.1)	874 (99.1)	442 (97.1)
Bilateral	21 (1.6)	3 (0.8)	18 (1.9)	8 (0.9)	13 (2.9)
ASA, n (%)					
1	52 (3.9)	19 (4.8)	33 (3.5)	46 (5.2)	6 (1.3)
2	611 (45.7)	194 (48.6)	417 (44.5)	434 (49.2)	177 (38.9)
3	412 (30.8)	109 (27.3)	303 (32.3)	229 (26.0)	183 (40.2)
4	8 (0.6)	2 (0.5)	6 (0.6)	4 (0.5)	4 (0.9)
Missing	254 (19.0)	75 (18.8)	179 (19.1)	169 (19.2)	85 (18.7)
Comorbidities, *n* (%)					
Low back pain	527 (39.4)	189 (47.4)	338 (36.0)	328 (37.2)	199 (43.7)
Other lower limb joint problem	500 (37.4)	169 (42.4)	331 (35.3)	319 (36.2)	181 (39.8)
Heart disease	496 (37.1)	139 (34.9)	357 (38.1)	326 (37)	170 (37.3)
Hypertension	857 (64.1)	224 (56.1)	633 (67.5)	519 (58.8)	338 (74.3)
Diabetes	343 (25.7)	66 (16.5)	277 (29.5)	173 (19.6)	170 (37.4)
Stomach or GIT condition	226 (16.9)	50 (12.5)	176 (18.8)	133 (15.1)	93 (20.4)
Lung disease	236 (17.7)	74 (18.5)	162 (17.3)	124 (14.1)	112 (24.6)
Renal failure	100 (7.5)	31 (7.8)	69 (7.4)	67 (7.6)	33 (7.3)
Liver disease	24 (1.8)	4 (1.0)	20 (2.1)	17 (1.9)	7 (1.5)
Neurological condition	75 (5.6)	26 (6.5)	49 (5.2)	50 (5.7)	25 (5.5)
Depression	203 (15.2)	56 (14.0)	147 (15.7)	124 (14.1)	79 (17.4)
Baseline HOOS/KOOS, mean (SD)					
Pain	31.5 (17.0)	31.1 (17.6)	31.7 (16.7)	32.5 (17.3)	29.3 (16.0)
Symptoms	34.7 (18.5)	33.5 (19.8)	35.2 (17.9)	35.5 (18.9)	33.1 (17.7)
Activities of daily living	33.1 (17.6)	31.4 (18.0)	33.9 (17.4)	34.4 (17.6)	30.5 (17.3)
Sport/Recreation	13.5 (19.2)	16.5 (18.8)	12.2 (19.3)	14.4 (18.7)	11.7 (20.3)
Quality of Life	18.6 (16.1)	19.1 (16.8)	18.4 (15.7)	19.4 (16.1)	17.0 (15.9)
Total	33.3 (16.9)	32.1 (17.6)	33.9 (16.5)	34.5 (17.0)	30.9 (16.5)
Baseline EQ-VAS, mean (SD)	57.8 (24.1)	55.9 (24.1)	58.7 (24.1)	58.6 (24.8)	56.4 (22.8)
Education, *n* (%)					
Certificate	32 (2.4)	10 (2.5)	22 (2.3)	22 (2.5)	10 (2.2)
Graduate Degree	34 (2.5)	12 (3.0)	22 (2.3)	27 (3.1)	7 (1.5)
No Formal Schooling	69 (5.2)	8 (2.0)	61 (6.5)	41 (4.6)	28 (6.2)
Post-Graduate Degree	20 (1.5)	6 (1.5)	14 (1.5)	14 (1.6)	6 (1.3)
Unknown	36 (2.7)	11 (2.8)	25 (2.7)	22 (2.5)	14 (3.1)
Year 10 or Equivalent	741 (55.4)	239 (59.9)	502 (53.5)	487 (55.2)	254 (55.8)
Year 12 or Equivalent	186 (13.9)	69 (17.3)	117 (12.5)	123 (13.9)	63 (13.8)
Year 6 or Equivalent	219 (16.4)	44 (11.0)	175 (18.7)	146 (16.6)	73 (16.0)
Preferred language, n (%)					
English	867 (64.8)	284 (71.2)	583 (62.2)	555 (62.9)	312 (68.6)
Other	470 (35.2)	115 (28.8)	355 (37.8)	327 (37.1)	143 (31.4)

*THA* total hip arthroplasty, *TKA* total knee arthroplasty, *BMI* body mass index, *n* number, *SD* standard deviation, *ASA* American Society of Anesthesiologists, *GIT* gastrointestinal tract, *HOOS* hip injury and osteoarthritis outcome score, *KOOS* knee injury and osteoarthritis outcome score, *EQ-VAS* EuroQol Visual Analogue Scale

**Table 3:** Baseline characteristics according to weight change in patients with BMI ≥ 30 kg/m^2^

	**Overall** **(*n* = 809)**	**Weight unchanged** **(*n* = 707)**	**Lost ≥ 5% baseline weight** **(*n* = 102)**
Gender, *n* (%)			
Female	551 (68.1)	476 (67.3)	75 (73.5)
Male	258 (31.9)	231 (32.7)	27 (26.5)
Age, years, mean (SD)	65.8 (9.26)	65.7 (9.38)	66.0 (8.44)
Baseline BMI, (kg/m^2^), mean (SD)	37.0 (5.75)	36.9 (5.69)	38.2 (6.03)
Surgery type, n (%)			
Total hip arthroplasty	193 (23.9)	171 (24.2)	22 (21.6)
Total knee arthroplasty	616 (76.1)	536 (75.8)	80 (78.4)
Unilateral	792 (97.9)	690 (97.6)	102 (100)
Bilateral	17 (2.1)	17 (2.4)	0 (0)
ASA, *n* (%)			
1	19 (2.3)	14 (2.0)	5 (4.9)
2	347 (42.9)	303 (42.9)	44 (43.1)
3	277 (34.2)	241 (34.1)	36 (35.3)
4	5 (0.6)	5 (0.7)	0 (0)
Missing	161 (19.9)	144 (20.4)	17 (16.7)
Comorbidities			
Low back pain	339 (41.9)	294 (41.6)	45 (44.1)
Other lower limb joint problem	316 (39.1)	268 (37.9)	48 (47.1)
Heart disease	315 (38.9)	270 (38.2)	45 (44.1)
Hypertension	563 (69.6)	491 (69.4)	72 (70.6)
Diabetes	258 (31.9)	222 (31.4)	36 (35.3)
Stomach or GIT condition	162 (20.0)	148 (20.9)	14 (13.7)
Lung disease	168 (20.8)	146 (20.7)	22 (21.6)
Renal failure	66 (8.2)	58 (8.2)	8 (7.8)
Liver disease	12 (1.5)	11 (1.6)	1 (1.0)
Neurological condition	47 (5.8)	40 (5.7)	7 (6.9)
Depression	131 (16.2)	114 (16.1)	17 (16.7)
Baseline HOOS/KOOS, mean (SD)			
Pain	30.9 (16.9)	30.3 (16.2)	35.1 (21.1)
Symptoms	33.9 (18.5)	33.4 (18.2)	37.7 (20.3)
Activities of daily living	31.7 (17.3)	31.3 (17.0)	34.7 (19.3)
Sport	12.6 (19.3)	12.3 (19.2)	14.3 (20.2)
Quality of life	17.9 (15.9)	17.7 (16.0)	19.9 (14.6)
Total	32.1 (16.6)	31.7 (16.2)	34.9 (19.1)
Baseline EQ-VAS, mean (SD)	56.8 (23.3)	57.3 (22.9)	53.5 (25.4)
Education, *n* (%)			
Certificate	20 (2.5)	15 (2.1)	5 (4.9)
Graduate Degree	18 (2.2)	16 (2.3)	2 (2.0)
No Formal Schooling	51 (6.3)	43 (6.1)	8 (7.8)
Post-Graduate Degree	12 (1.5)	10 (1.4)	2 (2.0)
Unknown	23 (2.8)	22 (3.1)	1 (1.0)
Year 10 or Equivalent	439 (54.3)	390 (55.2)	49 (48.0)
Year 12 or Equivalent	119 (14.7)	104 (14.7)	15 (14.7)
Year 6 or Equivalent	127 (15.7)	107 (15.1)	20 (19.6)
Preferred language, *n* (%)			
English	519 (64.2)	452 (63.9)	67 (65.7)
Other	290 (35.8)	255 (36.1)	35 (34.3)

*n* number, *SD* standard deviation, *BMI* body mass index, *ASA* American Society of Anesthesiologists, *GIT* gastrointestinal tract, *HOOS* hip injury and osteoarthritis outcome score, *KOOS* knee injury and osteoarthritis outcome score, *EQ-VAS* EuroQol Visual Analogue Scale

### Primary analysis according to BMI category

#### Postoperative complications

The incidence of postoperative complications is described in Tables 4 and 5. Overall, participants with BMI ≥ 35 kg/m^2^ experienced more complications to 6 months post-surgery compared to those with BMI < 35 kg/m^2^ though the difference was not significant (40% vs. 37%, *P* = 0.3). The types of complications are described in Table 1.

**Table 4:** Postoperative complications and patient-reported outcomes to 6 months post-surgery according to BMI category and arthroplasty type

	**Overall** **(*n* = 1,337)**	**THA** **(*n* = 399)**	**TKA** **(*n* = 938)**	**BMI < 35** **(*n* = 882)**	**BMI ≥ 35** **(*n* = 455)**
Complications, n (%)					
Acute to 6 months post-surgery	508 (38.0)	130 (32.6)	378 (40.3)	325 (36.8)	183 (40.2)
Major complications, n (%)					
Acute to 6 months post-surgery	143 (10.7)	45 (11.3)	98 (10.4)	94 (10.7)	49 (10.8)
Acute to discharge	68 (5.1)	22 (5.5)	46 (4.9)	45 (5.1)	23 (5.1)
From discharge to 6 months post-surgery	78 (5.8)	23 (5.8)	55 (5.9)	50 (5.7)	28 (6.2)
Oxford Score at 6 months post-surgery, (0–48), mean (SD)^l^					
Oxford Hip Score	41.7 (7.50)^a^	41.7 (7.50)^a^	-	41.5 (7.82)^b^	42.4 (6.25)^c^
Oxford Knee Score	38.2 (7.99)^d^	-	38.2 (7.99)^d^	38.6 (7.90)^e^	37.8 (8.13)^f^
EQ-VAS at 6 months post-surgery (0–100), mean (SD)^l^	75.1 (18.7)^g^	76.6 (18.1)^h^	74.5 (18.9)^i^	76.5 (18.1)^j^	72.5 (19.6)^k^
Patient-rated improvement from surgery, n (%)					
Much worse	22 (1.6)	3 (0.8)	19 (2.0)	13 (1.5)	9 (2.0)
A little worse	18 (1.3)	4 (1.0)	14 (1.5)	14 (1.6)	4 (0.9)
About the same	39 (2.9)	9 (2.3)	30 (3.2)	27 (3.1)	12 (2.6)
A little better	161 (12.0)	38 (9.5)	123 (13.1)	102 (11.6)	59 (13.0)
Much better	987 (73.8)	319 (79.9)	668 (71.2)	646 (73.2)	341 (74.9)
Missing	110 (8.2)	26 (6.5)	84 (9.0)	80 (9.1)	30 (6.6)

*THA* total hip arthroplasty, *TKA* total knee arthroplasty, *BMI* body mass index, *n* number, *SD* standard deviation, *EQ-VAS* EuroQol Visual Analogue Scale

^a^371 participants

^b^287 participants

^c^84 participants

^d^852 participants

^e^514 participants

^f^338 participants

^g^1225 participants

^h^372 participants

^i^853 participants

^j^803 participants

^k^422 participants

^l^Higher scores indicate better outcomes

**Table 5:** Postoperative complications and patient-reported outcome measures to 6 months post-surgery according to weight change in patients with BMI ≥ 30 kg/m^2^

	**Overall** ***(n* = 809)**	**Weight unchanged** **(*n* = 707)**	**Lost ≥ 5% baseline weight** **(*n* = 102)**
Complications, n (%)			
Acute to 6 months post-surgery	310 (38.3)	268 (37.9)	42 (41.2)
Major complications, n (%)			
Acute to 6 months post-surgery	86 (10.6)	74 (10.5)	12 (11.8)
Acute to discharge	42 (5.2)	36 (5.1)	6 (5.9)
From discharge to 6 months post-surgery	45 (5.6)	39 (5.5)	6 (5.9)
Oxford Score at 6 months post-surgery (0–48), mean (SD)^j^			
Oxford Hip Score	42.2 (6.98)^a^	42.1 (7.18)^b^	42.4 (5.17)^c^
Oxford Knee Score	37.8 (8.17)^d^	37.7 (8.08)^e^	38.2 (8.80)^f^
EQ-VAS at 6 months post-surgery (0–100), mean (SD)^j^	74.0 (18.8)^g^	74.0 (19.0)^h^	74.4 (18.0)^i^
Patient-rated improvement from surgery, n (%)			
Much worse	15 (1.9)	14 (2.0)	1 (1.0)
A little worse	11 (1.4)	10 (1.4)	1 (1.0)
About the same	21 (2.6)	19 (2.7)	2 (2.0)
A little better	106 (13.1)	89 (12.6)	17 (16.7)
Much better	598 (73.9)	524 (74.1)	74 (72.5)
Missing	58 (7.2)	51 (7.2)	7 (6.9)

*n* number, *SD* standard deviation, *EQ-VAS* EuroQol Visual Analogue Scale

^a^182 participants

^b^162 participants

^c^20 participants

^d^565 participants

^e^491 participants

^f^74 participants

^g^747 participants

^h^652 participants

^i^95 participants

^j^Higher scores indicate better outcomes

The results from unadjusted and adjusted logistic regression analyses of postoperative complications are presented in Tables 6 and 7, respectively. After adjusting for covariates, there was no significant difference in postoperative complications to 6 months post-surgery according to BMI category (OR 1.0, 95% CI 0.8–1.4, *P* = 0.8). The area under the ROC curve indicated a poor level of discrimination (0.62). Obesity (BMI ≥ 35 kg/m^2^) was not associated with higher odds of major complications in the acute postoperative period (surgery to discharge from hospital) and to 6 months post-surgery. A similar area under the ROC curve was obtained for the acute postoperative period (0.67) and post-acute period to 6 months post-surgery (0.64).

**Table 6:** Results of unadjusted regression models for postoperative complications and patient-reported outcome measures according to BMI and preoperative weight change

	**BMI ≥ 35 kg/m**^**2**^** vs. BMI < 35 kg/m**^**2**^			**Lost 5% of baseline weight vs. weight unchanged or increased**	
	**Overall sample** **(*n* = 1,337)**	**THA** **(*n* = 399)**	**TKA** **(*n* = 938)**	**Overall sample** **(*n* = 809)**	**TKA** **(*n* = 616)**
Overall complications to 6 months post-surgery OR (95% CI)	1.1 (0.9, 1.5), *P* = 0.3	1.4 (0.8, 2.2), *P* = 0.2	1.0 (0.8, 1.3), *P* = 0.9	1.1 (0.7, 1.7), *P* = 0.6	0.7 (0.4, 1.2), *P* = 0.2
Major complications to 6 months post-surgery OR (95% CI)	1.0 (0.7, 1.4), *P* = 1.0	1.1 (0.5, 2.2), *P* = 0.8	1.0 (0.6, 1.5), *P* = 0.9	1.1 (0.6, 2.1), *P* = 0.7	0.6 (0.2, 1.3), *P* = 0.2
Major complications to discharge OR (95% CI)	1.0 (0.6, 1.6), *P* = 1.0	1.7 (0.6, 4.1), *P* = 0.3	0.8 (0.4, 1.5), *P* = 0.6	1.2 (0.4, 2.6), *P* = 0.7	0.5 (0.1, 1.8), *P* = 0.4
Oxford Score at 6 months post-surgery Mean (95% CI)	-0.9 (-1.9, 0.02), *P* = 0.05	-	-	0.3 (-1.5, 2.0), *P* = 0.8	-
EQ-VAS at 6 months post-surgery Mean (95% CI)	-4.0 (-6.2, -1.8), *P* < 0.05	-	-	0.6 (-3.5, 4.6), *P* = 0.8	-
Patient-rated improvement from surgery OR (95% CI)	1.0 (0.7, 1.3), *P* = 0.9	-	-	0.9 (0.5, 1.5), *P* = 0.6	-

*BMI* body mass index, *n* number, *THA* total hip arthroplasty, *TKA* total knee arthroplasty, *OR* Odds Ratio, *CI* Confidence Interval, *EQ-VAS* EuroQol Visual Analogue Scale

**Table 7:** Results of adjusted regression models for postoperative complications and patient-reported outcome measures according to BMI and preoperative weight change

	**BMI ≥ 35 kg/m**^**2**^** vs. BMI < 35 kg/m**^**2**^			**Lost 5% of baseline weight vs. weight unchanged or increased**	
	**Overall sample** **(*n* = 1,337)**	**THA** **(*n* = 399)**	**TKA** **(*n* = 938)**	**Overall sample** **(*n* = 809)**	**TKA** **(*n* = 616)**
Overall complications to 6 months post-surgery OR (95% CI)	1.0 (0.8, 1.4), *P* = 0.8	1.5 (0.8, 2.8), *P* = 0.2	0.9 (0.6, 1.2), *P* = 0.4	1.1 (0.7, 1.8), *P* = 0.7	0.7 (0.4, 1.2), *P* = 0.2
Major complications to 6 months post-surgery OR (95% CI)	1.0 (0.6, 1.6), *P* = 1.0	1.0 (0.4, 2.4), *P* = 1.0	1.0 (0.6, 1.7), *P* = 1.0	1.2 (0.6, 2.5), *P* = 0.6	0.7 (0.2, 1.7), *P* = 0.4
Major complications to discharge OR (95% CI)	1.0 (0.5, 1.9), *P* = 1.0	1.1 (0.3, 3.6), *P* = 0.8	1.0 (0.5, 2.1), *P* = 1.0	1.7 (0.6, 4.4), *P* = 0.3	0.7 (0.1, 2.8), *P* = 0.7
Oxford Score at 6 months post-surgery Mean (95% CI)	-0.2 (-1.4, 0.9), *P* = 0.7, *R*^2^ = 0.04	-	-	0.6 (-1.3, 2.6), *P* = 0.5, *R*^2^ = 0.05	-
EQ-VAS at 6 months post-surgery Mean (95% CI)	-1.0 (-3.6, 1.6), *P* = 0.4, *R*^2^ = 0.08	-	-	0.7 (-3.5, 4.9), *P* = 0.7, *R*^2^ = 0.09	-
Patient-rated improvement from surgery OR (95% CI)	1.2 (0.8, 1.7), *P* = 0.5	-	-	0.9 (0.5, 1.8), *P* = 0.8	-

*BMI* body mass index, *n* number, *THA* total hip arthroplasty, *TKA* total knee arthroplasty, *OR* Odds Ratio, *CI* Confidence Interval, *EQ-VAS* EuroQol Visual Analogue Scale

Subgroup analysis was undertaken to investigate the association between complications and obesity according to arthroplasty type. Adjusted logistic regression analysis did not show a statistically significant difference in complication rate to 6 months post-surgery for TKA (OR 0.9, 95% CI 0.6–1.2, *P* = 0.4) or THA (OR 1.5, 95% CI 0.8–2.8, *P* = 0.2) according to BMI (Table 7). The area under the ROC curve was 0.62 for TKA and 0.63 for THA.

#### Patient-reported outcome measures

Mean Oxford Knee Scores and Oxford Hip Scores at 6 months post-surgery were similar regardless of BMI category. Participants with a BMI of 35 kg/m^2^ or greater scored lower on the EQ-VAS (mean 72.5 [SD 19.6] vs. 76.5 [SD 18.1], *P* = 0.001). Overall, over 70% of participants rated their operated joint as “much better” compared to before surgery. After adjusting for covariates, there was no statistically significant difference between groups according to BMI for any patient-reported outcome measure at 6 months post-surgery (Table 7). The area under the curve was 0.61 (poor) for the ROC curve of an association between BMI and patient-rated improvement from surgery.

### Secondary analysis according to preoperative weight loss

#### Postoperative complications

The presence of complications to 6 months post-surgery was similar in those losing 5% or more of their baseline weight compared to those who did not (41% vs. 38%, *P* = 0.7). After adjusting for covariates, preoperative weight loss was not associated with lower odds of overall (OR 1.1, 95% CI 0.7–1.8, *P* = 0.7) or major complications (OR 1.2, 95% CI 0.6–2.5, *P* = 0.6). The area under the ROC curve was 0.63 for overall complications and 0.62 for major complications. There was no statistically significant difference in complication rate to 6 months post-surgery for TKA according to preoperative weight loss (OR 0.7, 95% CI 0.4–1.2, *P* = 0.2). A separate analysis was not completed for THA due to the small sample size in the sub-sample.

#### Patient-reported outcome measures

Mean Oxford Hip and Knee Scores at 6 months post-surgery were similar regardless of preoperative weight change. After adjusting for covariates there was no statistically significant difference between groups according to preoperative weight change for any patient-reported outcome measure at 6 months post-surgery (Table 7). The area under the curve was 0.59 (poor) for the association between preoperative weight loss and patient-rated improvement from surgery.

## Discussion

This retrospective study did not identify an association between the presence of obesity (BMI ≥ 35 kg/m^2^) and complications or patient-reported outcomes 6 months post-surgery. Furthermore, the odds of developing a postoperative complication to 6 months post-surgery and worse patient-reported outcomes were not found to differ between patients with obesity who lost 5% or more of their baseline weight compared to those who did not.

Our findings are consistent with previous research which reported no association between postoperative complications and obesity [8–10], specifically obesity class I (BMI 30–34.99) and II (BMI 35–39.99) [34, 35]. However, the literature is conflicting with other studies suggesting obesity is associated with postoperative complications following TKA and THA, in particular, deep vein thrombosis [18], pulmonary embolism [5, 18], wound infection [18, 19] and revision surgery [36]. The lack of statistically significant findings may be explained by the use of BMI to measure obesity. While BMI accounts for an increase in body weight, it does not differentiate between the proportion of lean muscles to adipose tissue [37, 38]. Thus, healthy individuals with high muscle mass and a lower risk of postoperative complications may be classified as obesity. In fact, earlier studies concluded that obesity may be a protective factor associated with lower odds of early postoperative complications following TKA or THA [39–42]. Similarly, BMI does not distinguish between peripheral and visceral fat (the latter being fat associated with a greater risk of postoperative complications) [43]. Another possible explanation relates to an individual’s cardiometabolic risk profile. It is suggested that people with obesity and a normal cardiometabolic risk profile do not have a heightened risk for postoperative complications when compared to people with a healthy weight [42]. This may explain the lack of statistically significant findings in our study as our participants were enrolled in an optimization program (OACCP) while awaiting surgery. Participants with uncontrolled comorbidities (i.e., those with an increased risk of experiencing a postoperative complication based on the severity of their comorbidities) who did not proceed to surgery were not included in the dataset.

Concerning preoperative weight loss, our study did not demonstrate lower odds of experiencing a postoperative complication with 5% weight loss or more before undergoing TKA or THA. However, while there was no statistically significant association, there was a trend for lower odds of complications in participants undergoing TKA who lost 5% of their baseline weight. Our study may have been underpowered to detect a significant difference as only a small proportion of participants with obesity lost 5% of their baseline weight (*n* = 102). Thus, this issue requires further investigation. Evidence supporting the notion that preoperative weight loss reduces postoperative complications is derived from a recent study involving bariatric surgery as the weight loss intervention before TKA [44]. It provides the first confirmatory (and causal) evidence that significant preoperative weight loss in people with severe obesity (defined by the authors as BMI ≥ 35 kg/m^2^) does have a reduced risk for postoperative complications. Patients with severe obesity who underwent laparoscopic adjustable gastric banding and lost up to 20% of their baseline weight before TKA experienced fewer complications than the group who did not undergo bariatric surgery (14.6% vs. 36.6%, mean difference 22%; 95%CI 3.7%–40.3%, *P* = 0.02) [44].

Beyond the aforementioned study, there are also data from lower-level studies suggesting the benefit of weight loss may not be universal [45, 46]. For example, a retrospective study of 14,784 patients did not find a significant reduction in the risk of surgical site infections and 90-day readmission in patients with obesity who lost 5% of their body weight before TKA or THA [45]. The authors proposed that 5% weight loss preoperatively was insufficient to reduce complication risk. That is, the weight loss may not have been sufficient to change an individual’s BMI classification or improve their comorbidities. Similarly, a recent retrospective study investigating the effect of non-surgical preoperative weight loss in 1,589 patients undergoing THA found that complications were higher amongst those who lost weight [47]. It found that weight loss from a BMI >40kg/m2 to a BMI <40kg/m2 was associated with an increased risk of readmissions and complications. The authors hypothesized that patients who lost weight were at greater risk of postoperative complications, regardless of their weight loss, due to their comorbidity profile. It was also unclear whether the weight loss was intentional or occurred due to other circumstances such as illness which could have increased risk.

Regarding patient-reported outcome measures, our findings are consistent with other studies demonstrating similar improvement in patient-reported pain and function among people with and without obesity [36, 48], and that there is no significant association between BMI and patient-reported outcomes [36, 49]. However, the literature is conflicted with some studies reporting lower postoperative satisfaction levels following TKA [50] and worse patient-reported outcomes following TKA or THA in people with obesity [48, 51, 52]. The authors proposed that the experience of healthcare delivery and preoperative expectations influenced postoperative satisfaction in addition to BMI [50]. Concerning joint-specific or health-related patient-reported outcomes, the authors hypothesized that postoperative outcomes were limited by lower preoperative scores [52] and highlighted the importance of assessing the change score which revealed similar improvement in people with and without obesity [52].

## Strengths and limitations

This study has several strengths. First, it used prospectively collected data sourced from comprehensive databases. Second, unlike other retrospective studies investigating the association between obesity and postoperative outcomes up to 90 days post-surgery, this study evaluated outcomes to 6 months post-surgery. Thus, due to the longer follow-up period, this study captured arthroplasty-related complications that may occur beyond the commonly investigated 90-days postoperative period, such as revision surgery. Third, adjusted analysis was performed to account for the effect of comorbidities associated with postoperative complications in patients with obesity (e.g., diabetes and hypertension). Finally, to our knowledge, this is one of the few retrospective studies to analyze complication rates according to preoperative weight change that does not focus on bariatric surgery [45, 47].

The limitations of this study were related to the retrospective study design. Due to the non-random sampling, the results may have been influenced by unknown confounders regardless of our analyses that adjusted for known confounders. Furthermore, there were missing data due to loss to follow-up at 6 months post-surgery and errors in data entry. The proportion of patients with obesity who lost 5% or more of their baseline weight was small and thus regression analysis was not powered to detect an important difference. Additionally, the focus on 5% weight loss might have been too small to reduce complications. Finally, by adjusting for comorbidities, we may have hidden any association with obesity by adjusting for mediating variables. For example, if obesity (which is associated with higher rates of diabetes) affects postoperative complications because of the higher rate of diabetes, adjusting for diabetes will hide that effect.

## Conclusion

This retrospective study did not find a significant association between BMI and complications to 6 months or patient-reported outcomes following TKA or THA. Further, there was no significant association between preoperative weight loss in people with obesity and postoperative complications or patient-reported outcomes. Adequately powered studies are needed to confirm or deny our findings, with consideration given to whether 5% weight loss is sufficient to reduce post-operative complications.

### Supplementary Information

**Additional file 1.** Area under the receiver operating characteristic (ROC) curve and Hosmer and Lemeshow goodness of fit tests.

## Data Availability

Data relevant to this retrospective study are found in the article and supplementary file. Further data will be made upon request from the corresponding author.

## Abbreviations

BMI: Body Mass Index
OR: Odds Ratio
AOANJRR: Australian Orthopaedic Association National Joint Replacement Registry
TKA: Total knee arthroplasty
THA: Total hip arthroplasty
ACORN: Arthroplasty Clinical Outcomes Registry National
OACCP: Osteoarthritis Chronic Care Program
SWSLHD: South Western Sydney Local Health District
EQ-VAS: EuroQol Visual Analogue Scale
KOOS: Knee Injury and Osteoarthritis Outcome Score
HOOS: Hip Injury and Osteoarthritis Outcome Score
ASA: American Society of Anaesthesiology
ROC: Receiver operating characteristic
95% CI: 95% Confidence interval
