# Supplementary material for: The effect of body mass index and preoperative weight loss in people with obesity on postoperative outcomes to 6 months following total hip or knee arthroplasty: a retrospective study
Source: Arthroplasty. 2023 Oct 1;5:48. doi: 10.1186/s42836-023-00203-5 (PMC10544191; doi:10.1186/s42836-023-00203-5)
Supplement: Supplementary file 1 — Additional file 1. Area under the receiver operating characteristic (ROC) curve and Hosmer and Lemeshow goodness of fit tests. [file 42836_2023_203_MOESM1_ESM.docx]

**Additional file**

**Additional file 1. Area under the receiver operating characteristic (ROC) curve and Hosmer and Lemeshow goodness of fit tests.**

1. **Primary analysis investigating the association between BMI and outcomes.**

|  | **AUC** | **Hosmer and Lemeshow goodness of fit test** |
| --- | --- | --- |
| All postoperative complications to 6 months post-surgery | 0.62 | X-squared = 13.74, df = 8, p-value = 0.09 |
| Major complications to 6 months post-surgery | 0.64 | X-squared = 9.74, df = 8, p-value = 0.28 |
| Acute major complications | 0.67 | X-squared = 6.82, df = 8, p-value = 0.56 |
| Patient-rated improvement from surgery at 6 months post-surgery | 0.61 | X-squared = 2.41, df = 8, p-value = 0.97 |

AUC: area under curve

1. **Subgroup analysis investigating the association between BMI and outcomes in people undergoing total knee arthroplasty.**

|  | **AUC** | **Hosmer and Lemeshow goodness of fit test** |
| --- | --- | --- |
| All postoperative complications to 6 months post-surgery | 0.62 | X-squared = 4.20, df = 8, p-value = 0.84 |
| Major complications to 6 months post-surgery | 0.64 | X-squared = 9.01, df = 8, p-value = 0.34 |
| Acute major complications | 0.71 | X-squared = 9.09, df = 8, p-value = 0.33 |

AUC: area under curve

1. **Subgroup analysis investigating the association between BMI and outcomes in people undergoing total hip arthroplasty.**

|  | **AUC** | **Hosmer and Lemeshow goodness of fit test** |
| --- | --- | --- |
| All postoperative complications to 6 months post-surgery | 0.63 | X-squared = 12.88, df = 8, p-value = 0.12 |
| Major complications to 6 months post-surgery | 0.66 | X-squared = 5.48, df = 8, p-value = 0.71 |
| Acute major complications | 0.71 | X-squared = 4.58, df = 8, p-value = 0.80 |

AUC: area under curve

1. **Secondary analysis investigating the association between preoperative weight loss and outcomes.**

|  | **AUC** | **Hosmer and Lemeshow goodness of fit test** |
| --- | --- | --- |
| All postoperative complications to 6 months post-surgery | 0.63 | X-squared = 5.14, df = 8, p-value = 0.74 |
| Major complications to 6 months post-surgery | 0.62 | X-squared = 7.37, df = 8, p-value = 0.50 |
| Acute major complications | 0.66 | X-squared = 3.06, df = 8, p-value = 0.93 |
| Patient-rated improvement from surgery at 6 months post-surgery | 0.59 | X-squared = 7.87, df = 8, p-value = 0.45 |

AUC: area under curve

1. **Subgroup analysis investigating the association between preoperative weight loss and outcomes in people undergoing total knee arthroplasty.**

|  | **AUC** | **Hosmer and Lemeshow goodness of fit test** |
| --- | --- | --- |
| All postoperative complications to 6 months post-surgery | 0.64 | X-squared = 17.90, df = 8, p-value = 0.02 |
| Major complications to 6 months post-surgery | 0.64 | X-squared = 5.25, df = 8, p-value = 0.73 |
| Acute major complications | 0.73 | X-squared = 5.78, df = 8, p-value = 0.67 |

AUC: area under curve
